# Supplementary material for: Population Analysis Identifies 15 Multi-Variant Dominant White Haplotypes in Horses
Source: Animals (Basel). 2024 Feb 5;14(3):517. doi: 10.3390/ani14030517 (PMC10854588; doi:10.3390/ani14030517)
Supplement: Supplementary file 1 [file animals-14-00517-s001.zip › Supple Figure S1.pdf]

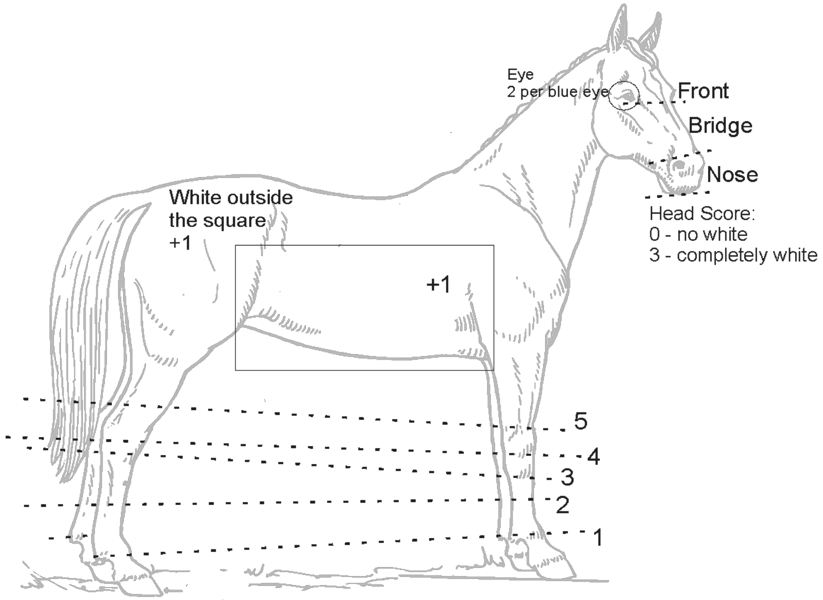

Supplementary Figure S1. Quantitative white spotting scoring diagram. We adapted the method as originally published to add two points for white on the body. Image adapted from Reider et al.
